# A three-years survey of microbial contaminants in industrial hemp inflorescences from two Italian cultivation sites

**DOI:** 10.1186/s42238-024-00241-z

**Published:** 2024-07-17

**Authors:** Gloria Spampinato, Francesco Candeliere, Alberto Amaretti, Roberta Paris, Massimo Montanari, Nino Virzì, Lorenzo Strani, Cinzia Citti, Giuseppe Cannazza, Maddalena Rossi, Stefano Raimondi

**Affiliations:** 1 Department of Life Sciences, University of Modena and Reggio Emilia, Via Campi 103, 41125 Modena, Italy; 2 Biogest-Siteia, University of Modena and Reggio Emilia, Via Amendola 2, 42122 Reggio Emilia, Italy; 3 CREA Research Centre for Cereal and Industrial Crops, Via Di Corticella 133, 40128 Bologna, Italy; 4 CREA Research Centre for Cereal and Industrial Crops, C.So Savoia 190, 95024 Acireale, CT Italy; 5 Department of Chemical and Geological Sciences, University of Modena and Reggio Emilia, Via Campi 103, 41125 Modena, Italy

**Keywords:** *Cannabis sativa* L., Agro-industrial waste, Herbal medicinal products, Yeasts, Moulds, Bacteria

## Abstract

**Background:**

The use of industrial *Cannabis sativa* L. for recreational, cosmeceutical, nutraceutical, and medicinal purposes has gained momentum due to its rich content of valuable phytochemicals, such as cannabidiol (CBD) and cannabigerol (CBG). However, there are concerns regarding the risk of microbial contamination in plants grown outside controlled environments. Microbes associated with hemp can be either epiphytes or endophytes and may pose a risk of infectious illness for humans.

**Methods:**

Seven Italian hemp genotypes, including Bernabeo, Carmagnola, Carmaleonte, Codimono, CS, Eletta Campana, and Fibranova, were cultivated in two distinct geographic locations, Catania and Rovigo, for three consecutive years from 2019 to 2021. Total aerobic microbes (TAMC), total combined yeasts/moulds (TYMC), the presence of bile-tolerant Gram-negative bacteria, and the absence of *Escherichia coli* and *Salmonella* spp. were evaluated and compared. The main phytocannabinoid content was measured and correlated with microbial contamination.

**Results:**

Most samples analyzed in this study did not meet the European Pharmacopoeia microbiological limits. The detection of potential pathogens, such as *E. coli* and *Salmonella* spp., in the samples indicates that the use of inflorescences may represent a possible source of infection. Microbial contamination varied among harvesting seasons and production sites, with agroclimatic conditions influencing microbial load and composition. The presence of potentially pathogenic bacteria was less associated with seasonal climate variability and more likely affected by sporadic contamination from external sources. CBD concentration exhibited a negative correlation with bile-tolerant Gram-negative bacteria and total yeasts/moulds levels. Samples with lower CBD content were more contaminated than those with higher CBD levels, suggesting a potential protective effect of this phytochemical on the plant.

**Conclusions:**

The threshing residues (inflorescences, floral bracts, and leaves) of industrial hemp varieties represent a valuable product and a source of beneficial phytochemicals that warrants further exploration. While post-harvest sterilization methods may reduce microbiological risks, they may also degrade heat- and light-sensitive bioactive phytochemicals. The most promising strategy involves implementing best agronomic practices to maintain healthy and uncontaminated cultures. Rigorous monitoring and quality certification protocols are essential to mitigate the microbiological risk associated with the consumption of hemp-derived products.

**Supplementary Information:**

The online version contains supplementary material available at 10.1186/s42238-024-00241-z.

## Background

Hemp (*Cannabis sativa* L.), also called “industrial hemp” has a long history, dating back to more than 6000 years ago (Moscariello et al., 2021). After a decline in the second half of the twentieth century, hemp cultivation had a revival in last decades, thanks to the selection of industrial varieties with a low concentration of the narcotic delta-9 THC. In Europe, the area dedicated to hemp cultivation has increased significantly from 20,540 hectares (ha) in 2015 to 33,020 ha in 2022, and the production increased of the 84.3% in the same period, with France accounting for more than 60% of EU production (https://agriculture.ec.europa.eu/farming/crop-productions-and-plant-based-products/hemp_en). A total of 115 hemp varieties are registered in the European Union (EU) Common Catalogue of Plant Varieties (accessed the 17th of March 2024). They are characterized by a CBD or CBG prevailing chemotype, with a THC content below the 0.3% threshold limit for hemp imposed by EU regulations (European Parliament and Council, 2013).

Hemp varieties are cultivated for various agricultural productions including fiber, seed, or dual purposes (fiber/seed). In addition to the primary agricultural products, hemp offers valuable by-products such as hurds, traditionally regarded as waste but holding significant potential for example within the context of biorefinery valorization. Hemp stands out as an exemplary model of circularity within the agricultural production chain, offering economic opportunities through the optimization of both primary and secondary resources (Moscariello et al., 2021). Among these resources, threshing residue, as termed by Calzolari et al. (2017), constitutes a mixture of leaves and inflorescences primarily comprising bracts and apical leaves obtained after seed harvest and cleaning. It accounts for approximately 30% (w/w) of the total hemp biomass (Matassa et al., 2020; Moscariello et al., 2021), with an estimated potential recovery from hemp cultivation in Northern Italy of up to 2 t ha^−1^ (Tang et al., 2016) and a yield of extracted CBD of up to 68 kg ha^−1^ (Calzolari et al., 2017). Consequently, this plant material could represent a source of profit by earning a space in the “Cannabis light” market and has garnered attention in recent years as a source of high-value bioactive compounds, including non-narcotic phytocannabinoids, flavonoids, and terpenes, making it attractive for various applications (Calzolari et al., 2017; Orlando et al. 2021; Pieracci et al. 2023; Cerrato et al., 2023).

The growing demand for inflorescences of *C. sativa* and the attempts in valorizing also the byproducts raise concerns about the quality assurance and associated risks of their consumption, either as such or in the form of derived products. These concerns have been compounded by the presence of contaminants of various types, including pesticides, fungicides, and other phytosanitary residues, heavy metals, carcinogens, mycotoxins, and microbial contaminants (Montoya et al. 2020). Infected materials containing bacteria or fungi may have adverse effects on the health of patients and consumers, particularly given the numerous reports of infections in immunocompromised subjects (Levi et al. 2019; Ruchlemer et al. 2015). Therefore, ensuring microbial quality is crucial for the supply of healthy products derived from *C. sativa*. Safeguard consumer health entails planning stringent quality control measures, complying with relevant regulatory standards, minimizing microbial contamination during cultivation and post-harvest handling, and implementing processing techniques to reduce microbial load (Jerushalmi et al. 2020).

This study aimed to assess the potential risks linked to the use of threshing residue derived from hemp seed cultivation. In particular, it focused on the potential exposure of consumers who directly utilize the inflorescence for recreational purposes, as well as the exposure of operators who come into contact with the material during processing phases, for example in the extraction plant of bioactive components. Specifically, the microbial contamination was examined in samples from seven Italian hemp genotypes (Bernabeo, Carmagnola, Carmaleonte, Codimono, CS, Eletta campana, and Fibranova) cultivated in two distinct geographical sites (Catania and Rovigo) for three consecutive years (2019–2021) and dried at ambient temperature in greenhouse facilities. According to the current European Pharmacopoeia, the level of total aerobic microbes (TAMC) and total combined yeasts/moulds (TYMC), the magnitude of bile-tolerant Gram-negative bacteria, and the presence or absence of *Escherichia coli* and *Salmonella* spp. were evaluated and compared. The content of the main phytocannabinoids was measured and correlated with the microbial contamination. Although the results do not encompass all possible microbial contaminations that can occur in various environments and across different hemp genotypes, they clearly underscore the potential risks associated with the use of this herbal matrix. Additionally, they highlight the significant impact of agroclimatic parameters on the microbial load.

## Methods

### Plant material, cultivation, harvesting, exsiccation of inflorescences, and sampling

Six hemp (*Cannabis sativa* L.) chemotype III varieties (Carmagnola, Carmaleonte, Codimono, CS, Eletta Campana, and Fibranova) and the chemotype IV (CBG rich) Bernabeo have been cultivated for three consecutive years (2019–2021) in two experimental farms (with three replicated parcels of 20–25 m^2^ each): “Busa Carrare”, located in Rovigo (RO; 45°04′45.4″N 11°45′57.3″E) and “ Libertinia”, located in Catania (CT; 37°32′25″N 14°34′41.0″E). They were all Italian varieties of the Italian/EU register of plant varieties, characterized by a prevalence of CBD (chemotype III) or CBG (chemotype IV) and a THC level below 0.2% (Table 1). The field experiment was carried out between April and October (according to the length of the life cycle of the hemp plants), with variations in sowing and harvesting dates differing between sites and among genotypes (Table 2). Nitrogen fertilization (40/60 units ha) was applied before sowing and irrigation was done only during seedling emergence as needed. Meteorological information (temperature and precipitation) was also collected, and the monthly averages are reported in Table 2. Female or monoecious inflorescences were taken at seed harvest time from three different parcels/repetitions for each genotype, dried at ambient temperature and with natural ventilation in local greenhouses for 48–72 h until 144 h for the later harvests of Rovigo. In both cultivation areas, for each harvesting season, the exsiccated biomass was trimmed and two samples of approximately 50 g (with inflorescences, floral bracts, and leaves) of all varieties were sealed in sterile bags, resulting in 42 duplicated samples, collected for microbiological contamination and phytocannabinoids content analyses.

**Table 1:** Key distinguishing traits of the different *Cannabis sativa* L. varieties utilized in the study

Variety	Sexual type	Main cannabinoid	Chemotype	Use	Geographical origin	Designation
Bernabeo^a^	dioeciuos	CBG	IV	Industrial	Italy	Cultivar^a^
CS	dioeciuos	CBD	III	Industrial	Italy	Cultivar
Carmagnola	dioeciuos	CBD	III	Industrial	Italy	Cultivar
Carmaleonte	monoecious	CBD	III	Industrial	Italy	Cultivar
Codimono	monoecious	CBD	III	Industrial	Italy	Cultivar
Eletta Campana	dioeciuos	CBD	III	Industrial	Italy	Cultivar
Fibranova	dioeciuos	CBD	III	Industrial	Italy	Cultivar

^a^ under registration in the National Register of Varieties under the name of “Felsinea”

**Table 2:** Weather parameters during the hemp growing seasons in Catania and Rovigo station: year of cultivation; sowing and harvesting date; average temperature (T °C) at the harvesting date; number of rainy days and total precipitations (mm) during the cultivation cycle

Cultivation sites	Year	Sowing date	Harvesting date	T (°C)	Rainy days	Rainfall (mm)
Catania	2019	02/04/2019	23/07/2019^a^	26.2	19	83.4
			31/07/2019^b^	28.0		
			28/08/2019^c^	27.3	22	118.3
	2020	09/04/2020	28/07/2020^a^	28.5	18	70.3
			04/08/2020^b^	28.6		
			18/09/2020^c^	23.9	30	187.0
	2021	02/04/2021	23/07/2021^a^	27.8	14	39.3
			31/07/2021^b^	30.3		
			28/08/2021^c^	27.8		
Rovigo	2019	19/04/2019	22/09/2019^a, b^	18.3	42	373.8
			03/10/2019^c^	16.0	48	396.2
	2020	23/04/2020	16/09/2020^a, b^	20.3	42	260.8
			08/10/2020c	13.1	51	295.0
	2021	26/04/2021	28/09/2021^a, b^	18.6	32	227.0
			12/10/2021c	12.0	35	247.4

^a^Carmaleonte (monoecius)

^b^Codimono (monoecius)

^c^Bernabeo, CS, Carmagnola, Eletta Campana, Fibranova (dioecius)

### Microbiological analyses

The composition of all media utilized for microbiological analysis is reported in Supplementary Table 1. All the materials, unless otherwise indicated, were purchased by Merck Life Science S.r.l. (Milan, Italy) or by Biolife Italia (Milan, Italy). In all the procedures microbial growth was obtained in static incubators (Binder GmbH, Tuttlingen, Germany) kept in aerobic conditions.

The microbial contamination of dried inflorescences was evaluated by culture-dependent methods in compliance with the quality parameters for herbal medicinal products required by the European Pharmacopoeia 9th Edition (Council of Europe 2019), that provide a detailed description of parameter, protocol, and material for the microbial analysis. In particular, the total aerobic microbial count (TAMC), the total combined yeasts/moulds count (TYMC), and the semiquantitative estimation (order of magnitude) of bile-tolerant Gram-negative Bacteria (BTGNB) were determined and expressed as colony forming units (CFU) *per* gram. The absence of *Escherichia coli* and *Salmonella* spp. (in 1 and 25 g, respectively) was assessed.

Twenty-five grams of sample were homogenized in 225 ml of casein soya-bean-digest broth (CSBDB) supplemented with 1 g/L of polysorbate for two minutes in a filter bag by using a blender. tenfold serial dilutions in CSBDB were prepared for TAMAC and TYMC estimation. Petri dishes of agarized CSBDB and Sabouraud-dextrose agar, the latter supplemented with chloramphenicol 50 mg/L, were seeded by surface-spread method, and incubated at 35 °C for 3 days and 25 °C for 5 days, respectively.

For semi-quantitative estimation of BTGNB, after a 2–3 h of incubation at 25 °C for bacterial resuscitation, the initial sample suspension was serially diluted in enterobacteria enrichment broth-Mossel and incubated at 35 °C for 24 h. Each dilution was plated in violet-red bile glucose agar and plates were incubated at 35 °C for 48 h. The growth of colonies represented a positive result for the corresponding dilution.

To test *E. coli* absence, 10 ml of suspension in CSBDB containing 1 g of sample was incubated at 35 °C for 24 h, then 1 mL of the enriched was seeded in 100 mL of MacConkey broth, incubated at 42 °C for 24 h, and the suspension plated on MacConkey agar, with plates incubated for 48 h at 35 °C.

To verify the absence of *Salmonella*, the sample suspension in CSBDB was incubated at 30 °C for 24 h, then 0.1 mL was diluted with 10 mL of Rappaport Vassiliadis *Salmonella* enrichment broth and the sample was incubated at 35 °C for 24 h. The enriched broth was then inoculated on a plate of xylose, lysine, and deoxycholate agar (XLDA). After incubation at 35 °C for 48 h, *Salmonella* contamination was revealed by the presence of well-grown red colonies, with or without a black center.

### Phytocannabinoids analyses

Phytocannabinoids were extracted from hemp biomass samples following the protocol reported in the monograph of *cannabis flos* included in the German Pharmacopoeia and adapted in our previous works (Tolomeo et al. 2022). Ethanol extraction was carried out on 500 mg of finely grounded hemp biomass in three cycles (20 mL, 12.5 mL, and 12.5 mL). The combined extracts were brought to 50 mL final volume with fresh EtOH in a volumetric flask. A 1 mL aliquot of the extract was centrifuged at 4000 × g for 5 min and filtered through a 0.45 µm regenerated cellulose filter, then diluted 10 times with mobile phase (acetonitrile/H_2_O, 60:40, *v/v*, 0.1% *v/v* formic acid).

Five µL of each sample were injected into the analytical apparatus Vanquish Core System (Thermo Fisher Scientific, Bremen, Germany) equipped with a binary pump, a vacuum degasser, a thermostated autosampler (4 °C) and column compartment (30 °C), a Poroshell 120 EC C18 column (100 × 3.0 mm I.D., 2.7 µm particle size, Agilent Technologies, Santa Clara, USA) and a diode array detector (DAD). The chromatographic parameters were adapted from a previously validated method with slight modifications (Tolomeo et al. 2022). The phytocannabinoids were separated with a constant flow rate of 0.5 mL/min, applying a gradient of acetonitrile from 60 to 95% in 15 min and an isocratic step at 95% acetonitrile held for 3 min, followed by a washing step of 4 min at 98% acetonitrile and a re-equilibration step at 60% acetonitrile for further 4 min. The analyses were acquired in the whole UV spectrum (190–400 nm) and chromatographic traces processed after filtration of the wavelength at 228 nm.

Quantification of phytocannabinoids was achieved using certified reference standard solution of cannabidiolic acid (CBDA), tetrahydrocannabinolic acid (THCA), cannabigerolic acid (CBGA), CBD, Δ^9^-THC, Δ^8^-THC, and CBG (1 mg/mL, Cerilliant, Merck Life Science S.r.l., Milan, Italy) diluted with mobile phase to get six non-zero calibration points at 0.1, 0.5, 1.0, 2.5, 5.0, and 10 µg/mL and analyzed with the same conditions used for the samples. Limit of detection (LOD) and limit of quantification were established at 0.03 and 0.1 µg/mL respectively. Linearity was assessed by the coefficient of determination (*R*^*2*^), which was greater than 0.997 for all cannabinoids (detailed information on calibration data in Supplementary Table 2). The back-calculated concentration was considered acceptable if it did not exceed 15% of the nominal value (and 20% for the LOQ). The raw data were processed with Chromeleon 7 (Thermo Fisher Scientific, Bremen, Germany) and the area of the peaks of the cannabinoids under investigations were used to calculate their concentration based on the respective calibration curves. The total content of phytocannabinoids was calculated using the formula: Total = 0.877*C + D, where C represents the amount of carboxylated species (either CBDA, Δ^9^- and Δ^8^-THC, or CBGA), and D represents the amount of decarboxylated species (either CBD, THC, or CBG).

### Statistical analysis

The variation of microbial contamination (TAMC, TYMC and BTGNB), as well as the presence or absence of *E. coli* and *Salmonella* spp., were inspected by considering three key factors: i) the different varieties, ii) the cultivation years, and iii) the geographical sites. This analysis was carried out using a Full Factorial Design of Experiments (DoE) (Leardi 2009), which estimates linear and interaction effects of the factors, considering all sources of information simultaneously. Forty-two samples, including all the possible combination between the different levels of each factor, i.e. seven varieties, three cultivation years (2019. 2020 and 2021) and two sites of cultivation (Rovigo and Catania), were randomly analyzed in duplicate to assess the significance of the factors with genuine replication (Box et al. 2005). The responses (TAM and TYM counts, BTGNB magnitude, *and E. coli* or *Salmonella* spp. presence/absence) were modeled using a Partial Least Squares (PLS) algorithm to simultaneously analyze all variations. The regression coefficients from PLS were used to estimate the effects of each factor on the response, identifying the influential parameters at high or low levels. Logistic regression (Christensen 2006). was employed within the DoE method to analyze the binary responses *of E. coli* and *Salmonella* spp.. Together, these statistical tools offer a methodology to identify optimal combinations of factors within a set of experiments, aiming to maximize classification accuracy (Lòpez et al., 2008).

The correlation between microbial contaminants and phytocannabinoid content was assessed using the Spearman coefficient, which is based on ranks. This robust measure does not rely on the assumption of a normal distribution of data.

Measures were reported as mean values ± standard deviation. The statistical analyses were performed using IBM SPSS Statistics 21.0 (IBM Corp., Armonk, NY, USA) and Design Expert v12 (Stat-Ease, Inc., Minneapolis, MN, USA).

## Results

A 3-year survey was conducted on microbial populations of *C. sativa* threshing residues obtained after seed collection from 7 hemp varieties cultivated at two different sites in North and South Insular Italy. Samples consisted of a mixture of inflorescences, floral bracts, leaves, and empty seeds remaining after seed selection. A total of 84 samples were collected and analyzed to assess the load of total aerobic microbes, yeast and molds and the presence of the most recurrent bacterial contaminants that pose a risk to human health (i.e., BTGNB, *E. coli*, and *Salmonella* spp.), as requested for herbal products by the European Pharmacopoeia.

**Fig. 1:**
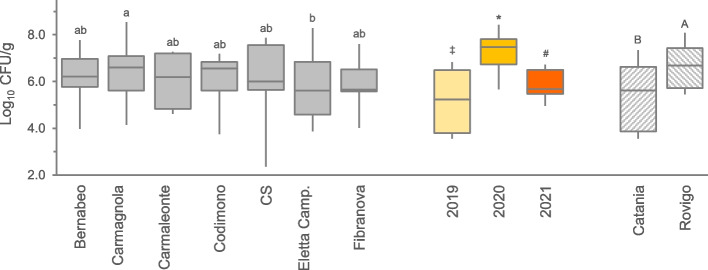
Total aerobic microbial count (TAMC) observed in the 7 hemp varieties cultivated in **a**) Catania and **b**) Rovigo during 3 years survey. The data were aggregated for variety, year, and location. Boxes indicate the median and 25th and 75th percentiles; whiskers indicate 10th and 90th percentiles Years legend: light yellow, 2019; dark yellow, 2020; orange, 2021. Common letters, symbols, or capital letters are used to indicate equivalent means among hemp varieties, cultivation years, and geographical sites, respectively

All the samples analyzed presented microbial contamination of mesophilic aerobes (TAMC), with a mean value of 6.0 ± 1.5 Log_10_ CFU/g for the whole dataset. Sporadic differences in microbial load between the varieties were observed during the 3 production seasons and in different production areas (Supplementary Fig. 1a, 1b). However, considering all samples *per* variety (Fig. 1), the observed average values were at most two-tier (*p* < 0.05), with Carmagnola and Eletta Campana having the most divergent values (6.4 ± 1.6 and 5.8 ± 1.6 Log_10_ CFU/g, respectively). Significant differences (*p *< 0.01) were observed between the three harvest seasons, with the highest load measured in 2020 (7.2 ± 1.2 Log_10_ CFU/g), followed by 2021 (5.8 ± 0.6 Log_10_ CFU/g), and 2019 (5.0 ± 1.5 Log_10_ CFU/g). TAMC was significantly affected by the production area as well, with samples from Rovigo showing higher contamination compared to the Catania ones (6.6 ± 1.6 and 5.4 ± 1.1 Log_10_ CFU/g, respectively). The DoE analysis enabled the assessment of how various factors interacted influencing microbiological parameters. Regarding TAMC, all second-order interactions were found to be significant. Notably, a distinct interaction was observed between the site and year of cultivation (Supplementary Fig. 1c). In the samples from Catania, TAMC values were significantly lower in 2019 compared to 2020 and 2021. Conversely, Rovigo exhibited generally higher TAMC values in 2020 and lower values in 2021. Additionally, there was a substantial disparity between the two cultivation sites in 2019, with Rovigo displaying significantly higher TAMC values than Catania. This variation may be attributed to the higher rainfall experienced at the northern site, particularly noteworthy in 2019.

**Fig. 2:**
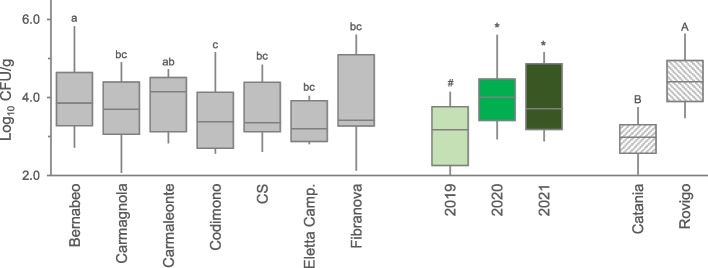
Total yeast and mould (TYMC) count observed in the 7 hemp varieties cultivated in **a**) Catania and **b**) Rovigo during 3 years survey. The data were aggregated for variety, year, and location. Boxes indicate the median and 25th and 75th percentiles; whiskers indicate 10th and 90th percentiles. Years legend: light green, 2019; green, 2020; dark green, 2021. Common letters, symbols, or capital letters are used to indicate equivalent means among hemp varieties, cultivation years, and geographical sites, respectively

Yeasts and molds were ubiquitous in *C. sativa* threshing residues, occurring at concentrations ranging from 2.1 to 6.5 Log_10_ CFU/g, with an average value of 3.7 ± 1.0 Log_10_ CFU/g. Similarly to TAMC, total yeast and mold count (TYMC) presented erratic values among varieties, in samples collected from Catania and Rovigo over three years (Supplementary Fig. 2a, 2b). The highest contaminations level was observed in Bernabeo and Carmaleonte reaching up to 4.0 ± 1.2 Log_10_ CFU/g; Fig. 2). The years of cultivation affected the load of yeasts and molds, although only 2019 showed a lower value (3.1 ± 1.0 Log_10_ CFU/g) compared to 2020 and 2021 (4.1 ± 0.9 and 4.0 ± 1.0 Log_10_ CFU/g, respectively). A notable difference in the average TYMC was registered among the production areas, with Catania characterized by a value 1.5 orders of magnitude lower than Rovigo (3.0 ± 0.6 and 4.5 ± 0.8 Log_10_ CFU/g, respectively). For TYMC, a significant interaction year vs variety was observed, with the factor site being significant just as main effect. In fact, Rovigo systematically shows higher TYMC values than Catania, regardless of year or variety (Supplementary Fig. 2c).

The microbial population in *C. sativa* inflorescences was generally marked by the presence of BTGNB, which were found in the vast majority of analyzed samples (Table 3). Plant material from Rovigo consistently showed loads greater than 10^3^ CFU/g, independent of the year (*p* > 0.05). Overall, production from Catania presented a lower BTGNB count compared to Rovigo (*p* < 0.01), although it was clearly affected by the harvesting season: the lower median value was observed in 2019 (< 10^2^ and < 10 CFU/g), followed by 2021 (< 10^3^ and < 10^2^ CFU/g), with only samples above 10^3^ CFU/g in 2020. No differences were observed between varieties (*p* > 0.05). Significant interactions were also observed for BTGNB. When comparing cultivation sites, differences were evident for the years 2019 and 2021, but not for 2020 (Supplementary Fig. 3). Additionally, a significant variation between the three years in Catania was observed, whereas Rovigo consistently maintained a BTGNB value < 103 CFU/g irrespective of the cultivation year.

**Table 3:** Semi-quantitative assessment of Bile Tolerant Gram-Negative Bacteria (BTGNB) and evaluation of absence of *E. coli* and *Salmonella* in 1 and 25 g of plant biomass, respectively

**BTGNB**	Catania*			Rovigo*		
	2019*	2020*	2021*	2019	2020	2021
Bernabeo	< 10^2^ and < 10	> 10^3^	< 10^3^ and < 10^2^	> 10^3^	> 10^3^	> 10^3^
Carmagnola	< 10^2^ and < 10	> 10^3^	< 10^3^ and < 10^2^	> 10^3^	> 10^3^	> 10^3^
Carmaleonte	< 10	> 10^3^	< 10^3^ and < 10^2^	> 10^3^	> 10^3^	> 10^3^
Codimono	< 10	> 10^3^	> 10^3^	> 10^3^	> 10^3^	> 10^3^
CS	< 10^2^ and < 10	> 10^3^	< 10^3^ and < 10^2^	> 10^3^	> 10^3^	> 10^3^
Eletta Campana	< 10^2^ and < 10	> 10^3^	< 10^3^ and < 10^2^	> 10^3^	> 10^3^	> 10^3^
Fibranova	< 10^2^ and < 10	> 10^3^	> 10^3^	> 10^3^	> 10^3^	> 10^3^
***E. coli***	Catania			Rovigo		
	2019	2020	2021*	2019	2020	2021*
Bernabeo	-	+	-	-	+	+
Carmagnola	+	-	+	+	-	+
Carmaleonte	-	-	+	-	-	+
Codimono	-	-	+	-	-	+
CS*	+	+	+	+	+	+
Eletta Campana	+	-	-	+	-	+
Fibranova	-	-	+	-	-	+
**Salmonella**	Catania			Rovigo		
	2019	2020*	2021	2019	2020*	2021
Bernabeo	-	-	-	-	-	-
Carmagnola	-	-	-	-	+	-
Carmaleonte	-	+	-	-	+	-
Codimono	-	-	-	-	-	-
CS	-	+	-	-	-	-
Eletta Campana	-	-	-	-	+	-
Fibranova	-	-	-	-	+	-

BTGNT: Bile Tolerant Gram-Negative Bacteria; x: cfu/g; + : presence; -: absence; *: significantly different groups (*p* < 0.05) among geographical area; cultivation year, and botanical variety

Half of the samples with BTGNB were also positive for *E. coli* (Table 3). The presence of this opportunistic pathogen was not linked to the geographical area of production (*p *< 0.05) and was highly recurrent in 2021 compared to 2019 and 2020 (*p* < 0.01). Salmonella was observed sporadically (Table 3), with 15% of samples testing positive and a significantly higher recurrence in 2020 (*p* < 0.01), which was the only harvest season characterized by the presence of this pathogen. No significant interactions among parameters were observed for *Salmonella* and *E. coli* contamination.

**Fig. 3:**
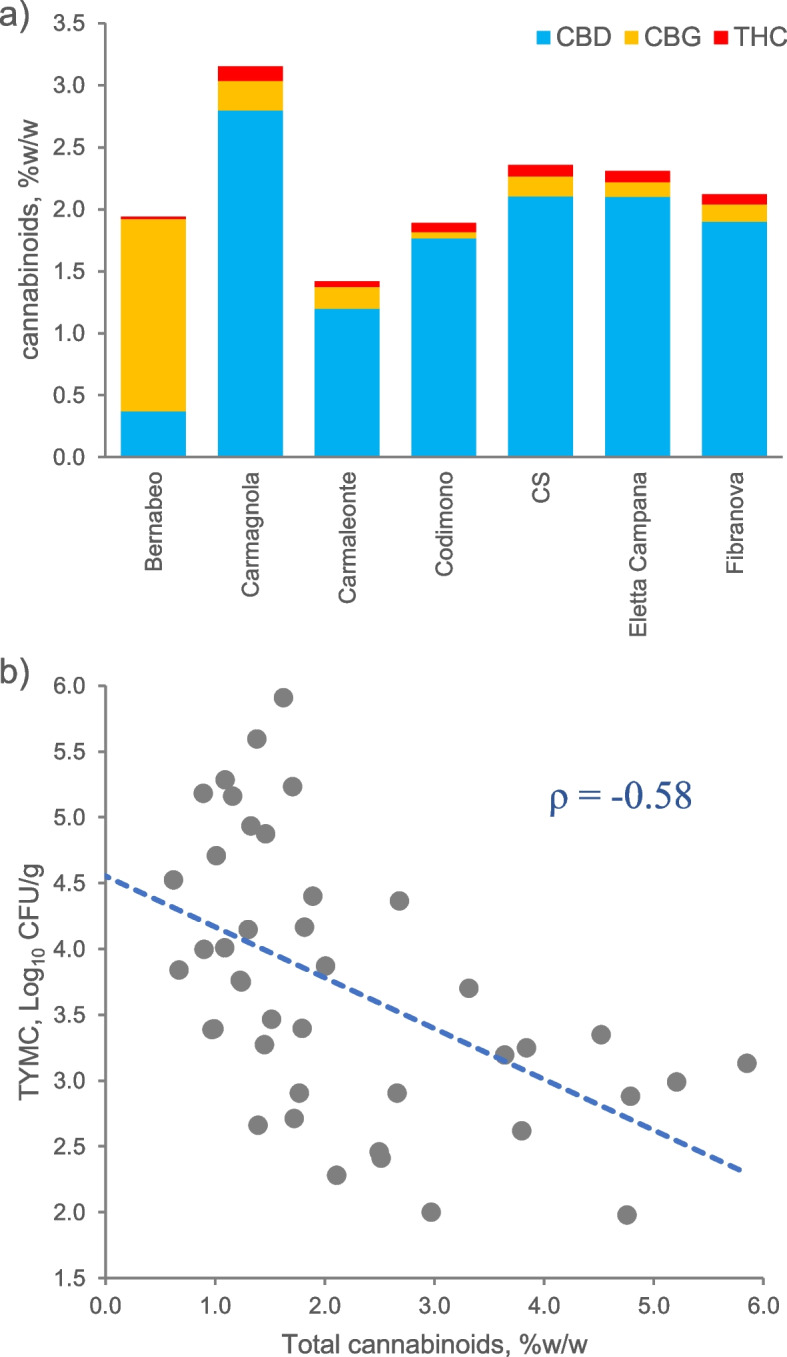
** a** Content of main phytocannabinoids (% w/w) in the analyzed threshing residues, obtained by HPLC–DAD analysis. Cyan, CBD; yellow, CBG; red, THC. **b** Spearman correlation between total yeast and moulds count (TYMC) and total cannabinoids ( *ρ*  = -0.58, *p*  < 0.01)

The HPLC–DAD analysis of the collected samples showed a wide variation in the total phytocannabinoids content, ranging from 0.62% to 5.85% w/w depending on the variety of hemp (Fig. 3a). As expected, CBD was found to be the most abundant cannabinoid, with a mean concentration of 1.75% w/w, followed by CBG (0.35% w/w). THC was detected in concentrations lower than 0.3% w/w and was negligible in most samples of the Bernabeo variety. Considering these mean values, and the yield of dried threshing residues of 350—450 kg ha^−1^ obtained in Catania, and 1200—1400 kg ha in Rovigo, we can estimate a CBD yield of 6—24 kg ha^−1^, and for CBG 1—4.9 kg ha^−1^.

The Spearman correlation coefficient was calculated to determine the relationship between the content of phytocannabinoids (total, CBD, CBG, and THC) and the levels of microbial contamination. A moderate but significant negative correlation (-0.58; *p* < 0.01) was observed between the content of total phytocannabinoids and TYMC (Fig. 3b). Also BTGNB were negatively correlated with the total phytocannabinoids content (-0.61, *p* < 0.01). Similar correlations were observed between CBD and both TYMC and BTGNB (-0.50 and -0.43, respectively). THC was found to inversely correlate only with TYMC (-0.42, *p* > 0.05). No significant correlation (*p* > 0.05) was observed between *E. coli*, *Salmonella* spp., and phytocannabinoid content.

## Discussion

The use of the floral part of industrial varieties of *Cannabis sativa* L. has gained increasing interest for cosmeceutical, nutraceutical, and medicinal purposes (Nadar et al., 2022; Zheng et al., 2022; Fortin et al., 2022). The phytocannabinoid profile of these inflorescences is usually characterized by the abundant presence of bioactive molecules such as CBD and cannabigerol (CBG) with a low or negligible amount of the psychotropic THC.

The utilization of plants or parts of them that are not produced in controlled environments poses the risk of microbial contamination, which can be transferred from the raw vegetal material to the final products, such as extracts, ointments, macerates, infusions, or the inflorescences themselves. *C. sativa*-associated microbes may be epiphytes or endophytes, residing on the surface or inside the plant tissues, respectively. Most endophytes originate from the rhizosphere, access plants via roots, and subsequently translocate through the xylem. Typically, they provide benefits for plant growth or are neutral (Reinhold-Hurek et al., 2011). Conversely, epiphytic microbes originate from several sources, such as dust, rain, irrigation water, or animal or human contact. Depending on their origin, they may present a concrete risk of infectious illness for humans (Brennan et al. 2022). To ensure a high level of microbiological quality, strict regulations have been introduced in many countries, including the European Pharmacopoeia (Ph. Eur.), which has set the acceptable count of total microbes (TAMC) at 50,000 colony forming units (CFU) per gram of herbal medicinal product and at 500 CFU/g for yeasts and molds (TYMC) (Ph. Eur. 5.1.8.C, 2023).

Ph. Eur. limits are very low compared to the counts observed in the samples of this study that reached a mean value of TAMC equal to 6.0 ± 1.5 Log_10_ CFU/g and a maximum charge higher than 8.0 Log_10_ CFU/g. Only a few samples harvested in Catania during the 2019 season met the quality criterion. Similarly, yeasts and moulds contamination exceeded the Ph. Eur. limits in most of the samples, with counts up to 6.0 Log_10_ CFU/g. These data are in accordance with previous information reported in the literature that deals with *C. sativa* varieties for medicinal use (Montoya et al. 2020), reporting that many species belonging to the genus *Penicillium*, *Aspergillus,* and *Fusarium* have been identified as endophyte colonizers. Such contamination poses a real threat in terms of fungal infection, as described for immunocompromised individuals (Ruchlemer et al. 2015), and exposure to carcinogenic mycotoxins (López-Ruiz et al. 2022). Although reports of bacterial infections caused by contaminated *C. sativa* are currently rare, the bacterial load observed in some samples, which was three orders of magnitude higher than the Ph. Eur. limit, raises significant concerns regarding the use of these threshing residues. Furthermore, bile-tolerant gram-negative bacteria were commonly detected in the analyzed samples, with sporadic detections of *E. coli* and *Salmonella* spp. These concerns are particularly relevant for industrial-scale cultivations, where preventing external contamination from animals, birds, and operators is challenging.The Ph. Eur. requires a count of BTGNB lower than 10^2^ CFU/g and the absence of *E. coli* and *Salmonella* spp. in 1 and 25 g of material, respectively. However, most of the analyzed samples did not meet these mandatory microbiological standards. Salmonellosis associated with marijuana consumption has been previously reported in the literature (Taylor et al. 1982), and the presence of *Salmonella* spp. in samples from both Catania and Rovigo, harvested in 2020, suggests that the recreational utilization of inflorescence from this year may have constituted a possible cause of infection by this pathogen. These concerns also extend to other cultivated hemp varieties harvested from different environments, where additional contaminations may occur, compromising the safety and quality of the resulting products and by-products.

Significantly different microbial contaminations were observed among both harvesting seasons and sites of production, which can be attributed to diverse agroclimatic conditions affecting microbial load and composition. Specifically, the number of rainy days, total precipitation, and mean temperature at the time of harvesting seemed to be important factors in microbial growth. A higher load of microbes (both TAMC and TYMC) was generally observed in Rovigo samples compared to Catania ones, and in 2020 compared to 2019 and 2021. During the three-year survey, Rovigo site was characterized by higher humidity (up to 51 rainfall days during cultivation) and lower temperatures at the harvesting (ranging from 12 to 20 °C) in respect to Catania site. These conditions may both have affected both the yield of threshing residues and the development of microbes on plants during cultivation and favored microbial growth during drying in greenhouses.

The presence of potentially pathogenic bacteria such as BTGNB, *E. coli,* and *Salmonella* spp., mainly ascribed to epiphyte microbiota, appeared to be less associated with seasonal climate variability and more likely affected by less predictable sporadic contaminations from external sources.

The antimicrobial effects of *C. sativa* essential oils or extracts have been extensively studied and reported (Nissen et al., 2010; Karas et al. 2020). Metabolic fingerprinting has revealed the presence of more than 480 compounds, including numerous terpenes and nearly 180 phytocannabinoids (Fischedick et al., 2010). Although the growth inhibitory effect is likely due to synergism between several compounds, isolated phytocannabinoids have been found to exhibit potent antimicrobial activity. For example, they have been shown to be effective against Gram-positive pathogens, such as *Staphylococcus aureus*, *Streptococcus pneumoniae*, and *Clostridioides difficile*, as well as a subset of Gram-negative bacteria, including *Neisseria gonorrhoeae* (Appendino et al. 2008; Blaskovich et al. 2021). On the other hand, recent studies suggest that phytocannabinoids concentration can be stimulated in the plant through symbiotic and/or mutualistic relationships with endophytes (Taghinasab et al., 2020).

In the analyzed samples, it was observed that the amounts of phytocannabinoids did not appear to have a correlation with the overall microbial load. However, a negative moderate correlation was found between the total phytocannabinoid content and the level of contamination by both BTGNB and yeast/molds. Specifically, the concentration of CBD exhibited a negative correlation with BTGNB and TYMC levels, whereas THC only showed a negative correlation with TYMC. Samples with lower CBD content, such as Carmaleonte and Bernabeo, were much more contaminated than those with higher CBD levels, such as Eletta Campana and CS. These findings suggest a possible protective effect of this phytochemical, but further investigation is needed to confirm this hypothesis.

## Conclusions

The cultivation of *Cannabis sativa* L. under sterile conditions is not a practical solution for industrial hemp production. Our study is the first to report the monitoring of microbial contaminants in industrial hemp accessions over three consecutive harvesting seasons. The vast majority of threshing residues (comprising inflorescences, floral bracts, and leaves) failed to meet the standards outlined in the European Pharmacopoeia (Ph. Eur.), highlighting a significant microbiological risk. This result can reasonably be extended to other industrial-scale outdoor cultivations. Direct consumption for recreational purposes or manipulation prior to solvent treatments in processing facilities potentially exposes consumers and operators to contaminants, leading to asthma, allergies, and infections. These hazards are exacerbated in instances where cultivation and production do not occur under controlled conditions, a common scenario for hemp. Attention must be paid to crop wholesomeness, particularly in humid areas or seasons, as well as to the exsiccation procedure. The cultivation of varieties with high CBD content might reduce microbial contamination levels, likely due to its antimicrobial activity and a protective role in the plant. While post-harvesting sterilization methods, such as ultraviolet irradiation or autoclaving, may reduce microbiological risks, they may also degrade the bioactive phytochemicals which are sensitive to heat and light (Jerushalmi et al. 2020). Therefore, the most promising strategy is to implement best agronomic practices to maintain healthy and uncontaminated cultures. Nevertheless, rigorous monitoring and quality certification protocols are essential to mitigate the microbiological risk associated with the consumption of hemp-derived products.

### Supplementary Information

Supplementary Material 1.

## Data Availability

The authors declare that the data supporting the findings of this study are available within the paper and its Supplementary Information files. Should any raw data files be needed in another format they are available from the corresponding author upon reasonable request.

## Abbreviations

BTGNB: Bile-tolerant Gram-negative Bacteria
CBD: cannabidiol
CBDA: Cannabidiolic acid
CBG: Cannabigerol
CBGA: Cannabigerolic acid
CFU: Colony-forming units
CSBDB: Casein soya-bean-digest broth
CT: Catania
EU: European Union
HIV: Human immunodeficiency virus
HPLC-DAD: High Performance Liquid Chromatography-Diode Array Detector
I.D.: Internal Diameter
LOD: Limit of detection
LOQ: Limit of quantification
RO: Rovigo
spp.: Species
TAMC: Total aerobic microbes
THC: Δ^9^-Tetrahydrocannabidiol
THCA: Tetrahydrocannabinolic acid
TYMC: Total combined yeasts/moulds

## References

[CR1] Appendino G, Gibbons S, Giana A, Pagani A, Grassi G, Stavri M, Smith E, Rahman MM (2008). Antibacterial cannabinoids from Cannabis sativa: a structure-activity study. J Nat Prod.

[CR2] Blaskovich MAT, Kavanagh AM, Elliott AG, Zhang B, Ramu S, Amado M, Lowe GJ, Hinton AO, Pham DMT, Zuegg J, Beare N, Quach D, Sharp MD, Pogliano J, Rogers AP, Lyras D, Tan L, West NP, Crawford DW, Peterson ML, Callahan M, Thurn M (2021). The antimicrobial potential of cannabidiol. Commun Biol.

[CR3] Box GE, Hunter JS, Hunter WG. Statistics for experimenters. In Wiley series in probability and statistics. Hoboken, NJ: Wiley; 2005. ISBN: 978-0-471-71813-0.

[CR4] Brennan FP, Alsanius BW, Allende A (2022). Harnessing agricultural microbiomes for human pathogen control. ISME COMMUN.

[CR5] Calzolari D, Magagnini G, Lucini L, Grassi G, Appendino G, Amaducci S. High added-value compounds from Cannabis threshing residues. Ind Crops Prod. 2017;108. 10.1016/j.indcrop.2017.06.063.

[CR6] Cerrato A, Biancolillo A, Cannazza G, Cavaliere C, Citti C, Laganà A, Marini F, Montanari M, Montone CM, Paris R, Virzì N, Capriotti AL. Untargeted cannabinomics reveals the chemical differentiation of industrial hemp based on the cultivar and the geographical field location. Anal Chim Acta. 2023;1278. 10.1016/j.aca.2023.341716. 10.1016/j.aca.2023.341716 37709459

[CR7] Christensen R (2006). Log-linear models and logistic regression.

[CR8] Council of Europe. European Pharmacopoeia: supplement 9.7. 9th ed. Strasbourg, Council of Europe; 2019. 5.1.8 microbiological quality of herbal medicinal products for oral use and extracts used in their preparation; p. 6474.

[CR9] European Parliament and Council. Regulation (EU) No 1308/2013 establishing a common organisation of the markets in agricultural products. Brussels. 2013.

[CR10] Fischedick  JT, Hazekamp A, Erkelens T,  Choi  YH, Verpoorte R (2010). Metabolic fingerprinting of Cannabis sativa L., cannabinoids and terpenoids for chemotaxonomic and drug standardization purposes. Phytochemistry.

[CR11] Fortin D, Marcellin F, Carrieri P, Mancini J, Barré T. Medical cannabis: toward a new policy and health model for an ancient medicine. Front Public Health 2022; 10. 10.3389/fpubh.2022.904291. 10.3389/fpubh.2022.904291 PMC9197104 35712276

[CR12] Jerushalmi S, Maymon M, Dombrovsky A, Freeman S (2020). Effects of cold plasma, gamma and e-beam irradiations on reduction of fungal colony forming unit levels in medical cannabis inflorescences. J Cannabis Res.

[CR13] Karas JA, Wong LJM, Paulin OKA, Mazeh AC, Hussein MH, Li J, Velkov T (2020). The Antimicrobial Activity of Cannabinoids. Antibiotics (basel).

[CR14] Leardi R (2009). Experimental design in chemistry: A tutorial. Anal Chim Acta.

[CR15] Levi  ME, Montague BT, Thurstone C, Kumar D, Huprikar SS, Kotton CN (2019). Executive Committee of the Infectious Diseases Community of Practice of the American Society of Transplantation. Marijuana use in transplantation: A call for clarity. Clin Transplant.

[CR16] López F, Valiente JM, Prats JM, Ferrer A (2008). Performance evaluation of soft color texture descriptors for surface grading using experimental design and logistic regression. Pattern Recogn.

[CR17] López-Ruiz R, Marín-Sáez J, GarridoFrenich A, Romero-González R (2022). Recent applications of chromatography for analysis of contaminants in cannabis products: a review. Pest Manag Sci.

[CR18] Matassa S, Esposito G, Pirozzi F, Papirio S. Exploring the Biomethane Potential of Different Industrial Hemp (Cannabis sativa L.) Biomass Residues. Energies. 2020;13(13):3361. 10.3390/en13133361.

[CR19] Montoya Z, Conroy M, Vanden Heuvel BD, Pauli CS, Park SH (2020). Cannabis Contaminants Limit Pharmacological Use of Cannabidiol. Front Pharmacol.

[CR20] Moscariello C, Matassa S, Esposito G, Papirio S (2021). From residue to resource: the multifaceted environmental and bioeconomy potential of industrial hemp (*Cannabis sativa* L.). Resour Conserv Recycl.

[CR21] Nadar RM et al. Cannabis-Based Cosmetic Products and Their Uses. In: Agrawal, D.C., Kumar, R., Dhanasekaran, M. (eds) Cannabis/Marijuana for Healthcare. 2022; Springer, Singapore. 10.1007/978-981-16-8822-5_13.

[CR22] Nissen L, Zatta A,  Stefanini I, Grandi  S, Sgorbati B, Biavati  B, Monti  A (2010). Characterization and antimicrobial activity of essential oils of industrial hemp varieties (*Cannabis sativa* L.). Fitoterapia.

[CR23] Orlando G, Adorisio S, Delfino D, Chiavaroli A, Brunetti L, Recinella L, Leone S, D'Antonio M, Zengin G, Acquaviva A, Antico M, Angelini P, Angeles Flores G, Venanzoni R, Tacchini M, Di Simone SC, Menghini L, Ferrante C (2021). Comparative Investigation of Composition, Antifungal, and Anti-Inflammatory Effects of the Essential Oil from Three Industrial Hemp Varieties from Italian Cultivation. Antibiotics (basel).

[CR24] Pieracci Y, Fulvio F, Isca V, Pistelli L, Bassolino L, Montanari M, Moschella A, Flamini G, Paris R (2023). The phenological stage of hemp inflorescences affects essential oil yield and its chemical composition. Ind Crops Prod.

[CR25] Reinhold-Hurek B, Hurek T (2011). Living inside plants: bacterial endophytes. Curr Opin Plant Biol.

[CR26] Ruchlemer R, Amit-Kohn M, Raveh D, Hanuš L (2015). Inhaled medicinal cannabis and the immunocompromised patient. Support Care Cancer.

[CR27] Taghinasab M, Jabaji S (2020). Cannabis Microbiome and the Role of Endophytes in Modulating the Production of Secondary Metabolites: An Overview. Microorganisms.

[CR28] Tang K, Struik PC, Yin X, Thouminot C, Bjelková M, Stramkale V, Amaducci S. Comparing hemp (Cannabis sativa L.) cultivars for dual-purpose production under contrasting environments. Ind Crops Prod. 2016;87. 10.1016/j.indcrop.2016.04.026.

[CR29] Taylor DN, Wachsmuth IK, Shangkuan YH (1982). Salmonellosis associated with marijuana: a multistate outbreak traced by plasmid fingerprinting. N Engl J Med.

[CR30] Tolomeo F, Russo F, Kaczorova D, Vandelli MA, Biagini G, Laganà A, Capriotti AL, Paris R, Fulvio F, Carbone L, Perrone E, Gigli G, Cannazza G, Citti C (2022). Cis-Δ^9^-tetrahydrocannabinolic acid occurrence in Cannabis sativa L. J Pharm Biomed Anal.

[CR31] Zheng H,  Chen B, Rao J (2022). Nutraceutical potential of industrial hemp (Cannabis sativa L.) extracts: physicochemical stability and bioaccessibility of cannabidiol (CBD) nanoemulsions. Food Funct.

