# Supplementary material for: A three-years survey of microbial contaminants in industrial hemp inflorescences from two Italian cultivation sites
Source: J Cannabis Res. 2024 Jul 17;6:31. doi: 10.1186/s42238-024-00241-z (PMC11253326; doi:10.1186/s42238-024-00241-z)
Supplement: Supplementary file 1 — Supplementary Material 1. [file 42238_2024_241_MOESM1_ESM.docx]

**A three-years survey of microbial contaminants in industrial hemp inflorescences from two Italian cultivation sites**

Gloria Spampinato^1^, Francesco Candeliere^1^, Alberto Amaretti^1, 2^, Roberta Paris^3^, Massimo Montanari^3^, Nino Virzì^4^, Lorenzo Strani^5^, Cinzia Citti^1^, Giuseppe Cannazza^1^, Maddalena Rossi^1, 2^_,_ and Stefano Raimondi^1, 2,*^

^1^ Department of Life Sciences, University of Modena and Reggio Emilia, via Campi 103, 41125, Modena, Italy

^2^ Biogest-Siteia, University of Modena and Reggio Emilia, via Amendola 2, 42122, Reggio Emilia, Italy

^3^ CREA Research Centre for Cereal and Industrial Crops, via di Corticella 133, 40128, Bologna, Italy

^4^ CREA Research Centre for Cereal and Industrial Crops, C.so Savoia 190, 95024, Acireale (CT), Italy

^5^ Department of Chemical and Geological Sciences, University of Modena and Reggio Emilia, via Campi 103, 41125, Modena, Italy

* corresponding author: stefano.raimondi@unimore.it

**Supplementary material**

**Supplementary Table 1.** Composition of media utilized for microbiological analyses.

Total aerobic microbial count (TAMC)

| **Casein Soya Bean Digest Agar (CSBDA)** | **Concentration (g/L)** |
| --- | --- |
| Pancreatic hydrolysate of casein | 15 |
| Papic hydrolysate of soy | 5 |
| NaCl | 5 |
| Agar | 15 |
| pH: 7.3 |  |

| **Casein Soya Bean Digest Broth (CSBDB)** | **Concentration (g/L)** |
| --- | --- |
| Pancreatic hydrolysate of casein | 17 |
| Papic hydrolysate of soy | 3 |
| NaCl | 5 |
| K_2_HPO_4_ | 2.5 |
| Glucose monohydrate | 2.5 |
| pH: 7.3 |  |

Total combined yeasts/moulds count (TYMC)

| **Sabouraud Dextrose Agar (SDA)** | **Concentration (g/L)** |
| --- | --- |
| Glucose | 40 |
| Mixture of pectic hydrolyzate of animal tissues and pancreatic hydrolyzate of casein (1: 1) | 10 |
| Agar | 15 |
| Chloramphenicol | 20 µg/mL |
| Ampicillin | 100 µg/mL |
| pH: 5.6 |  |

Semiquantitative estimation of bile-tolerant Gram-negative Bacteria (BTGNB)

| **Enterobacteria Enrichment Broth Mossel (EBM)** | **Concentration (g/L)** |
| --- | --- |
| Pancreatic gelatin hydrolysate | 10 |
| Glucose monohydrate | 5 |
| Dehydrated bovine bile | 20 |
| KH_2_PO_4_ | 2 |
| Na_2_HPO_4_ *2H_2_O | 8 |
| Brilliant green | 15 mg/L |
| pH: 7.2 |  |

| **Violet Red Bile Glucose Agar (VRBGA)** | **Concentration (g/L)** |
| --- | --- |
| Yeast extract | 3 |
| Pancreatic gelatin hydrolysate | 7 |
| Bile salts | 1.5 |
| NaCl | 5 |
| Glucose monohydrate | 10 |
| Agar | 15 |
| Neutral red | 30 mg/L |
| Crystal violet | 2 mg/L |
| pH: 7.4 |  |

Absence of *Escherichia coli*

| **MacConkey Broth** | **Concentration (g/L)** |
| --- | --- |
| Pancreatic gelatin hydrolysate | 20 |
| Lactose monohydrate | 10 |
| Dehydrated ox bile | 5 |
| Bromocresol violet | 10 mg/L |
| pH: 7.3 |  |

| **MacConkey Agar** | **Concentration (g/L)** |
| --- | --- |
| Pancreatic gelatin hydrolysate | 17 |
| Lactose monohydrate | 10 |
| Meat peptones and casein | 3 |
| NaCl | 5 |
| Bile salts | 1.5 |
| Agar | 13.5 |
| Neutral red | 30 mg/L |
| Crystal violet | 1 mg/L |
| pH: 7.1 |  |

Absence of *Salmonella* spp.

| **Rappaport Vassiliadis Salmonella Enrichment Broth (RVSEB)** | **Concentration (g/L)** |
| --- | --- |
| Soy peptone | 4.5 |
| MgCl_2_ * 6H_2_O | 29 |
| NaCl | 8 |
| K_2_HPO_4_ | 0.4 |
| KH_2_PO_4_ | 0.6 |
| Malachite green | 0.036 |
| pH: 5.2 |  |

| **Xylose, Lysine, Deoxycholate Agar (XLDA)** | **Concentration (g/L)** |
| --- | --- |
| Xylose | 3.5 |
| L-lysine | 5 |
| Lactose monohydrate | 7.5 |
| Sucrose | 7.5 |
| NaCl | 5 |
| Yeast extract | 3 |
| Phenol red | 80 mg/L |
| Agar | 13.5 |
| Sodium deoxycholate | 2.5 |
| Sodium thiosulfate | 6.8 |
| Ferric ammonium citrate | 0.8 |
| pH: 7.4 |  |

**Supplementary Table 2.** Calibration data of each analyzed phytocannabinoid (retention time, slope, intercept, and coefficient of determination R^2^.

| **Cannabinoid** | **RT (min)** | **Slope** | **Intercept** | **R2** |
| --- | --- | --- | --- | --- |
| CBDA | 6.46 | 0.288 | 0.013 | 0.999 |
| CBGA | 6.89 | 0.197 | 0.008 | 0.999 |
| CBG | 7.23 | 0.145 | 0.009 | 0.998 |
| CBD | 7.42 | 0.149 | 0.012 | 0.997 |
| CBN | 9.64 | 0.265 | 0.024 | 0.997 |
| Δ9-THC | 10.84 | 0.125 | 0.008 | 0.998 |
| Δ8-THC | 11.06 | 0.082 | 0.006 | 0.998 |
| THCA | 12.27 | 0.216 | 0.014 | 0.997 |

**Supplementary Figure 1**

Total aerobic microbial count (TAMC) observed in the 7 hemp varieties cultivated in a) Catania and b) Rovigo during 3 years survey (light yellow, 2019; dark yellow, 2020; orange, 2021). For each bar, the standard deviation and statistical significance (p < 0.05) are given. Common letters or symbols are used to indicate equivalent means among hemp varieties and cultivation years, respectively. c) Interaction effect between year and site (green, Rovigo; red, Catania) factors on TAMC response, in DoE multivariate analysis.

**Supplementary Figure 2**

Total yeast and mould count (TYMC) observed in the 7 hemp varieties cultivated in a) Catania and b) Rovigo during 3 years survey (light green, 2019; green, 2020; dark green, 2021). For each bar, the standard deviation and statistical significance (p < 0.05) are given. Common letters or symbols are used to indicate equivalent means among hemp varieties and cultivation years, respectively. c) c) Interaction effect between year and site (green, Rovigo; red, Catania) factors on TYMC response, in DoE multivariate analysis.

**Supplementary Figure 3**

Interaction effect between year and site (green, Rovigo; red, Catania) factors on bile-tolerant Gram-negative Bacteria (BTGNB) response, in DoE multivariate analysis.
